# Oligonol suppresses lipid accumulation and improves insulin resistance in a palmitate-induced in HepG2 hepatocytes as a cellular steatosis model

**DOI:** 10.1186/s12906-015-0709-1

**Published:** 2015-06-16

**Authors:** Jae-Yeo Park, Younghwa Kim, Jee Ae Im, Hyangkyu Lee

**Affiliations:** Department of Clinical Nursing Science, Yonsei University College of Nursing, 120-752 Seoul, Korea; Nursing Policy and Research Institute, Biobehavioral Research Center, Yonsei University, 120-752 Seoul, Korea; Department of Emergency Medical Technology, Kyuongil University, 712-701 Gyeongsan, Kyungbook Korea; Sport and Medicine Research Center, INTOTO Inc., 401 Dawoo BD, 90-6 Daeshin-Dong, 120-160 Seodaemun-Gu, Seoul Korea

**Keywords:** Oligonol, Hepatic steatosis, de novo fatty acid synthesis, Inflammation, Insulin resistance

## Abstract

**Background:**

Oligonol is a low molecular weight form of polyphenol polymers derived from lychee fruits. Several studies suggest that Oligonol has an anti-obesity effect. Since obesity is tightly associated with insulin resistance, we investigated a possible remission effect of Oligonol on lipid accumulation and insulin resistance in human hepatic HepG2 cells.

**Methods:**

HepG2 cells were treated with palmitate for 24 h to induce cellular hepatic steatosis and insulin resistance. The cells were then treated with Oligonol at subtoxic concentrations and examined for lipid metabolism, cytokine production, and insulin signaling using quantitative RT-PCR and western blot analysis.

**Results:**

Oligonol treatment reversed the palmitate-induced intracellular lipid accumulation, down regulated the expression of lipogenic genes, and up-regulated genes for fatty acid degradation. Oligonol restored insulin sensitivity, as was determined by the phosphorylation states of IRS-1. Oligonol also inhibited STAT3-SOCS3 signaling and increased AMPK phosphorylation in HepG2 cells.

**Conclusion:**

Oligonol treatment improved palmitate-induced cellular steatosis and insulin resistance in HepG2 cells with concomitant reduction of inflammatory cytokines and decrease in STAT3-SOCS3 and AMPK-mTOR pathways. Oligonol may have beneficial effects in lipid metabolism and insulin resistance in the liver.

## Background

The mammalian liver controls the metabolic homeostasis for carbohydrates, lipids, and proteins. Functional failure of the organ is detrimental to an organism. Non-alcoholic fatty liver disease (NAFLD) is a broadly defined term for fatty liver-related diseases [1, 2]. An early form of NAFLD is hepatic steatosis, characterized by the deposition of triglycerides in the liver as lipid droplets. Hepatic steatosis makes patients prone to more serious forms of NAFLD, such as non-alcoholic steatohepatitis (NASH) and liver cancer. Currently, there are not many therapeutic options for these diseases as the pathologies are not well understood [1].

The “two-hit hypothesis” has been proposed to understand the pathogenesis of NAFLD [3]. The “first hit” involves the deposition of triglycerides (TGs) and free fatty acids in hepatocytes, leading to the development of simple steatosis and insulin resistance, and the “second hit” involves hepatic injury, inflammation, and fibrosis, which are closely associated with oxidative stress in the liver [4, 5]. Lipid peroxidation, production of inflammatory cytokines, and mitochondrial dysfunction are the attributes of inflammatory stress [6]. To study hepatic steatosis in vitro, a cellular hepatic model was previously established by treating human HepG2 hepatocarcinoma cells with free fatty acids (FFAs) [7]. Upon treatment with palmitate (PA), a saturated fatty acid, these cells show excess accumulation of cytosolic lipids [7], activation of lipogenic genes [8], and development of insulin resistance [9, 10], which are the cellular aspects of hepatic steatosis.

Oligonol is a low molecular weight oligomer of long-chain polyphenols, typically proanthocyanidin found in various fruits, including lychees [11, 12]. Polyphenols derived from fruits and vegetables are known to function as anti-inflammatory and chemopreventive agents on various human diseases [13–15]. Despite its relatively recent introduction, Oligonol has been shown to display potential therapeutic effects on cancer, neurological, or metabolic disorders [11, 16]. Particularly, a randomized clinical trial showed that Oligonol reduces abdominal circumference and visceral fat, results in weight loss, and improves insulin resistance in healthy obese individuals [17]. Therefore, we hypothesized that Oligonol induced anti-inflammatory mechanisms and prevented the development of hepatic steatosis and liver damage.

The present study evaluated the effects of Oligonol in reducing hepatic steatosis in PA-treated HepG2 cells. This study showed that Oligonol suppressed de novo fatty acid synthesis and inflammatory cytokine production, which are associated with the inhibition of STAT3–SOCS3 and AMPK-mTOR.

## Methods

### Cell culture and Oligonol treatment

Human HepG2 cell line (a gift from Dr. E.-J. Lee at Yonsei University College of Medicine, Korea) was maintained in Hyclone Dulbecco’s Modified Eagle’s Medium (DMEM; Thermo Scientific, Rockford, IL, USA) containing 10 % fetal bovine serum, 100 U/mL penicillin, and 100 μg/mL streptomycin. Cells were cultured in 5 % CO_2_ at 37 °C. Oligonol (KCF Korea Co., Seoul, Korea) was dissolved in double-distilled H_2_O (ddH_2_O), diluted in DMEM, and used to treat HepG2 cells. The final quantity of solvent did not exceed 0.1 % of culture media for all experiments.

### MTT assay

HepG2 cells were grown in 96-well plate with different concentrations of Oligonol for 24 or 48 h. Cell viability was determined by colorimetry using the 3-(-4, 5-dimethylthiazol-2-yl)-2, 5-diphenyltetrazolium bromide (MTT) (USB corporation, Cleveland, OH, USA; Sigma-Aldrich, Oakville, Canada). Insoluble formazan crystals were dissolved in DMSO and the absorbance was measured at 490 nm in a microplate reader (Molecular Devices, Mountain View, CA, USA).

### Preparation and treatment of sodium palmitate in HepG2 cells

After reaching 80 % confluence, the cells were cultured with fetal bovine serum-free medium containing 5 % bovine serum albumin (BSA) overnight in a 6-well plate. Sodium palmitate (Sigma-Aldrich) was conjugated to fatty acid free-BSA (Sigma-Aldrich). Briefly, 69.6 mg sodium palmitate was dissolved in 1 ml sterile water by alternating heating and vortexing at 70 °C to obtain 250 mM stock solution. Immediately after the dissolution of PA, the stock solution was added to serum-free DMEM containing 5 % fatty acid-free BSA to obtain 250 μM PA solution. This solution was used to treat the cells [18]. Cells were treated with 200 μL of 250 μM PA or with different concentrations of Oligonol (1, 5, or 10 μg/ml) for 24 h. Cells cultured in a medium containing 5 % BSA were used as a control.

### Oil Red O staining

HepG2 cells grown in 6-well plates were collected, washed with phosphate buffered saline, and fixed with 4 % paraformaldehyde for 1 h. Cells were rinsed with ddH_2_O, dipped in 60 % isopropanol for a few seconds, stained with Oil Red O (Sigma Chemical, St. Louis, MO, USA) for 10 min, and rinsed with ddH_2_O few times to remove the excess stain. Cell nuclei were stained with hematoxylin for a few seconds and washed with ddH_2_O. Pictures were taken using an Axiovert 40CFL microscope (Olympus, Tokyo, Japan). For quantification of Oil Red O-based steatosis, HepG2 cells were cultured in a 96-well microplate at 5000 cells/well with PA plus/minus Oligonol for 24 h and Oil Red O staining was performed as described above. After washing and drying completely, 100 μL of extraction solution was added to each well and the mixtures were incubated for 10 min, followed by gentle vibration to release Oil Red O. The extraction solution was then transferred to another 96-well plate for measurement of optical density (OD) at 500 nm by microplate reader (Versamax; Molecular Devices Corporation, CA, USA). Tests were performed in triplicates.

### Quantitative real-time PCR (qRT-PCR)

One microgram of total RNA purified with RNeasy Mini Kit (Qiagen, Valencia, CA, USA) was used to synthesize cDNA (Quanti Tech Reverse Transcription Kit; Qiagen). cDNA was amplified using SYBR Premix Ex Taq and gene-specific primers (Table 1) in Takara Thermal Cycler Dice Real-Time system (Otsu, Shiga, Japan) (95 °C for 5 s, 58 °C for 10 s, and 72 °C for 20 s for 40 cycles). All reactions were performed in triplicates and the data were normalized to glyceraldehyde 3-phosphate dehydrogenase (GAPDH).

**Table 1:** Primer sequences used in this study

Genes	Forward primer	Reveres primer	Tm (C°)
ACC	GAAGTCAGAGCCACGGCACA	GGCAATCTCAGTTCAAGCCAGTC	62
FAS	AGCACTGCCTTCGGTTCAGTC	AAGAGCTGTGGAGGCCACTTG	62
SREBP1c	CTCCGGCCACAAGGTACACA	GAGGCCCTAAGGGTTGACACAG	60
HSL	CAAGTGTGTCAGCGCCTATGCT	GGGCTTTCTGGTCTGAGTTGGA	64
PPARγ	CTTGTGAAGGATGCAAGGGT	ATACAAATGCTTTGCCAGGG	62
CPT-1a	GGAATGAAATTCCCACTGTCTGTC	CAGTTCAGCCATCGCTGTTGTA	60
HADHα	ACTTCCAAAGACACCAGTGCTTCA	GGCGCAAGACACCTGGTAGTATAGA	60
LCAD	CCAGCTGCATGAAGCGAAAC	GCTGAACTCTGGCATCCACATAA	60
UCP2	TGTGCCCTTACCATGCTCCA	AGGGCTCGTTTCAGCTGCTC	60
CYP2E1	ATGTCTGCCCTCGGAGTGA	GATGTCCTTCCAGGTAGGTCC	58
CYP4A11	AGGAGCTCCAACAGGACCAG	CCTGATGGGTGAAGGCACAC	68
PPARα	TCCTGAGCCATGCAGAATTTAC	AGTCTAAGGCCTCGCTGGTG	60
TNFα	GACAAGCCTGTAGCCCATGTTGTA	CAGCCTTGGCCCTTGAAGA	60
IL-6	GTGGAGATTGTTGCCATCAACG	CAGTGGATGCAGGGATGATGTTCTG	60
MCP-1	AGCAGCAAGTGTCCCAAAGA	GGTGGTCCATGGAATCCTGA	60
STAT3	GGAATCCCGTGGGTTGCTTAC	TTGAATGCAGTGGCCAGGAC	60
SOCS3	CAGGAATGTAGCAGCGATGGAA	CCTGTCCAGCCCAATACCTGA	60
GAPDH	TGAACGGGAAGCTCACTGG	TCCACCACCCTGTTGCTGTA	58–64

### Western blot analysis

Proteins were extracted from the cells using extraction buffer [50 mM Tris HCl, pH 8.0, 5 mM EDTA, 150 mM NaCl, 0.5 % sodium deoxycholate, 1 % Nonidet P-40, 0.1 % SDS, 1 mM PMSF, 1 mM NaF, 1 mM NaVO_4_, and protease inhibitor cocktail (Roche, Germany). Protein extracts were first separated on 10 % polyacrylamide gels and electrophoretically transferred onto polyvinylidene fluoride membrane (Gelman Laboratory, Ann Arbor, MI, USA). After blocking, the membranes were incubated with a primary antibody, and then with horseradish peroxidase-conjugated IgG (Santa Cruz Biotechnology, Santa Cruz, CA, USA). Blots were developed using ECL Detection Kit (Amersharm Pharmacia, UK). Primary antibodies to ACC, pACC, HSL, pHSL, FAS, STAT3, pSTAT3, IRS-1, pIRS-1(Ser363), AMPK, pAMPK, mTOR, pmTOR, S6K, pS6K were from Cell Signaling Technology (Denver, MA, USA), PPARγ and pIRS(Tyr632) were from Santa Cruz Biotechnology (Santa Cruz, CA, USA), SREBP-1 was from BD Biosciences (San Jose, CA, USA), and β-actin was from Sigma (St. Louis, MO, USA). Results of western blotting were normalized with those obtained for β-actin. Protein bands were quantified using densitometry with accompanying software (ImageJ).

### Free fatty acids and glycerol assay

After treatment HepG2 cells with a various concentration of Oligonol in the presence of PA, free fatty acid and free glycerol contents in the cell supernatants was quantified using a free fatty acid or glycerol quantification kit according to the manufacturer’s instructions (Biovision Inc., Milpitas, CA, USA). Released free fatty acid or glycerol was quantified at 570 nm on a 96-well plate reader (Molecular devices, Mountain View, CA, USA).

### Statistical analysis

Data are presented as mean ± SEM. Differences between the means of each group were analyzed using Student’s *t* test or one-way ANOVA test with Dunnett’s multiple comparison test. *P* values < 0.05 were considered significant. The statistical software package Prism 5.0 (GraphPad Software, La Jolla, CA, USA) was used.

## Results

### Oligonol attenuated PA-induced intracellular lipid deposition in HepG2 cells

**Fig. 1:**
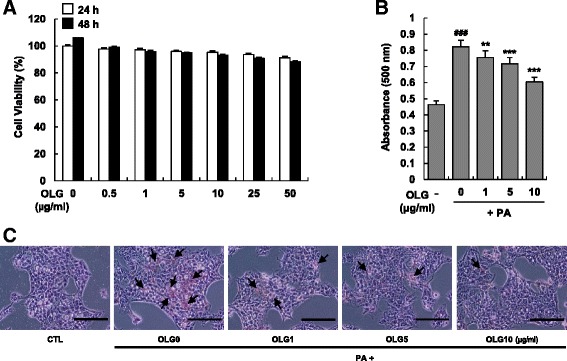
Oligonol attenuated PA-induced increase in intracellular lipid content in HepG2 cells. (**a**) Oligonol cytotoxicity measurement. HepG2 cells were incubated with various concentrations of Oligonol (OLG; ranging from 0 to 50 μg/ml) for 24 or 48 h, and cell viability was measured by MTT assay. (**b** and **c**) Cells were treated with 250 μM of palmitate (PA) in the absence or presence of Oligonol (ranging from 1 to 10 μg/ml) for 24 h, and stained with Oil-red O to visualize the intracellular lipid contents. (**b**) Lipid contents were quantified by its OD value at 500 nm. Data were presented as mean ± SEM of three independent experiments; each performed in triplicates. ^###^ *P* < 0.005 vs. vehicle-treated control; ** *P* < 0.01, and *** *P* < 0.005 vs. palmitate-treated cells (PA). (**c**) Micrographs of phase contrast microscopy. Control (CTL) is vehicle treated cells. *Scale bar* is 100 μm

To assess the cytotoxic effects of Oligonol on HepG2 cells, the cells were treated with various concentrations of Oligonol (0–50 μg/ml) for 24 and 48 h. Cell viability did not decrease below 90 % of the control level after 48 h, as determined by the MTT assay, indicating that the tested concentrations of Oligonol did not grossly affect cell growth and viability (Fig. 1a). Because the tested concentrations did not induce cytotoxicity, 1, 5, or 10 μg/ml of Oligonol was used for further studies.

Next, we investigated whether Oligonol inhibited PA-induced intracellular lipid accumulation in HepG2 cells [7]. The cells were incubated in 250 μM PA and various concentrations of Oligonol for 24 h, and intracellular lipid content was measured using oil red O staining. PA-treated HepG2 cells showed higher oil red O staining (Fig. 1b and c) than vehicle-treated control cells, indicating higher TG content. Oligonol suppressed TG accumulation in HepG2 cells in a dose-dependent manner.

### Oligonol suppressed lipogenesis and enhanced lipolysis in PA-treated HepG2 cells

**Fig. 2:**
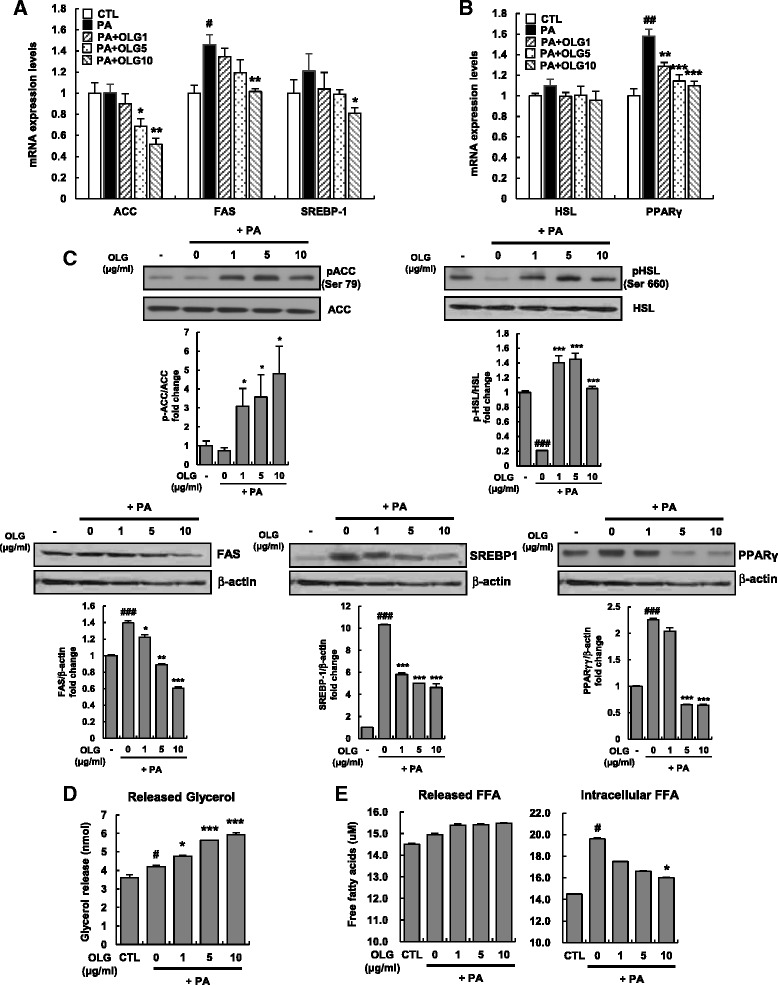
Oligonol suppressed lipogenesis while enhancing lipolysis in PA-treated HepG2 cells. HepG2 cells were treated with 250 μM of PA in the absence or presence of Oligonol (ranging from 1 to 10 μg/ml) for 24 h. (**a**) ACC, FAS, and SREBP-1c mRNA levels, (**b**) HSL and PPARγ mRNA levels were measured by qRT-PCR. Values were normalized to GAPDH and presented as mean ± SEM of three independent experiments; each performed in triplicates. *White bars* represent HepG2 cells treated with vehicle, and *black bars* represent HepG2 cells treated with PA. (**c**) Protein levels and phosphorylation states for these five genes were examined by Western blot analysis with appropriate antibodies. β-actin is a loading control. Graphs represent densitometric analysis of Western blots. (**d** and **e**) Released glycerol and FFAs into the culture medium and intracellular FFA contents were quantified by its OD value at 570 nm. Data were presented as mean ± SEM of three independent experiments; each performed in triplicates. ^#^ *P* < 0.05, ^##^ *P* < 0.01, ^###^ *P* < 0.005 vs. vehicle-treated control (CTL); ^*^ *P* < 0.05, ^**^ *P* < 0.01, and ^***^ *P* < 0.005 vs. palmitate-treated cells (PA)

Next, we examined the mechanisms underlying the inhibition of hepatic TG synthesis by Oligonol. Fatty acid synthesis begins with acetyl-CoA and involves the addition of two carbon units to increase the length of the fatty acid chain. Acetyl-CoA carboxylase (ACC) converts acetyl-CoA to malonyl-CoA, which is a rate-limiting step in lipogenesis [19]. Fatty acid synthase (FAS) utilizes acetyl-CoA, malonyl-CoA, and NADPH, and elongates fatty acids chain to form PA [20]. Therefore, we examined the key enzymes and transcription factors involved in lipid metabolism in HepG2 cells. Oligonol suppressed the mRNA and protein expression of ACC, FAS, sterol regulatory element-binding protein-1 (SREBP-1*)*, and peroxisome proliferator-activated receptor-γ (PPARγ) in a dose-dependent manner (Fig. 2a, b, and c), suggesting that Oligonol suppresses de novo fatty acid synthesis in HepG2 cells. Because Oligonol did not affect the mRNA levels of hormone sensitive lipase (HSL), we evaluated whether Oligonol regulated the activity of HSL by analyzing the levels of phosphorylated HSL in total cell lysates by performing western blotting. Phosphorylation of HSL at serine 660 was attenuated in PA-treated HepG2 cells, but all the 3 concentrations of Oligonol increased HSL phosphorylation. Further, we also found that phosphorylation of ACC at serine 79 was significantly increased in Oligonol-treated HepG2 cells (Fig. 2c).

Because Oligonol modulated the expression of genes involved in hepatic lipid metabolism, we examined whether the reduction in lipid accumulation was associated with lipolysis. HepG2 cells were incubated with Oligonol for 24 h, and the amounts of FFAs and glycerol in the culture medium were measured by performing a colorimetric assay. Oligonol increased the secretion of glycerol into the medium in a dose-dependent manner (Fig. 2d). In contrast, Oligonol did not increase the secretion of FFA into the medium and significantly decreased the intercellular levels of FFA (Fig. 2e).

### Oligonol increased fatty acid oxidation in PA-treated HepG2 cells

**Fig. 3:**
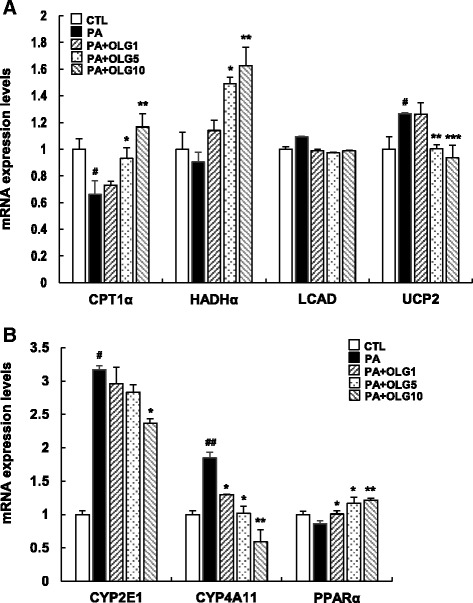
Oligonol increased lipid oxidation in PA-treated HepG2 cells. HepG2 cells were treated with 250 μM of PA in the absence or presence of Oligonol (ranging from 1 to 10 μg/ml) for 24 h. (**a**) CPT1a, HADHα, LCAD, and UCP2 mRNA levels, (**b**) CYP2E1, CYP4A11, and PPARα mRNA levels were measured using qRT-PCR. Values were normalized to GAPDH. Data were presented as mean ± SEM of three independent experiments; each performed in triplicates. *White bars* represent HepG2 cells treated with vehicle, and *black bars* represent HepG2 cells treated with PA. ^#^ *P* < 0.05, ^##^ *P* < 0.01 vs. vehicle-treated control (CTL); ^*^ *P* < 0.05, ^**^ *P* < 0.01, ^***^ *P* < 0.005 vs. palmitate-treated cells (PA)

Next, we examined whether Oligonol enhanced the degradation of fatty acids through β-oxidation. PA notably decreased the mRNA level of carnitine palmitoyltransferase 1a (CPT1a) in HepG2 cells, whereas Oligonol increased the mRNA level of CPT1a in a dose-dependent manner (Fig. 3a). PA had a similar effect on the level of long-chain L-3-hydroxyacyl-coenzyme A dehydrogenase α (HADHα). However, Oligonol had no effect on long-chain acyl-CoA dehydrogenase (LCAD) under the same condition. We also found that Oligonol attenuated PA-induced increase in the mRNA level of uncoupling protein 2 (UCP2), suggesting that Oligonol has antioxidant properties and protects hepatocytes from PA-induced lipotoxicity.

ω-Oxidation, an alternative pathway of fatty acid metabolism, is activated upon failure of β-oxidation. Previous studies have shown that expression of the cytochrome P450 enzymes CYP2E1 and CYP4A11 is increased in patients with NAFLD [21] and that PA significantly increases the level of CYP2E1 [22]. Therefore, we examined whether Oligonol inhibited the expression of these enzymes. Treatment of HepG2 cells with PA increased the mRNA levels of CYP2E1 and CYP4A11 by up to three fold; however, these levels were markedly reduced after Oligonol treatment.

Transcription factor PPARα directly controls various steps of hepatic lipid metabolism, including expression of CPT1a and HADHα [23]. Because these genes are upregulated by Oligonol, we determined whether expression of PPARα was also upregulated by Oligonol. Oligonol increased the mRNA level of PPARα in a dose-dependent manner (Fig. 3b), indicating that Oligonol improves fatty acid catabolism at the transcriptional level at least in part by regulating the expression of PPARα.

### Oligonol suppressed PA-induced inflammation in HepG2 cells

**Fig. 4:**
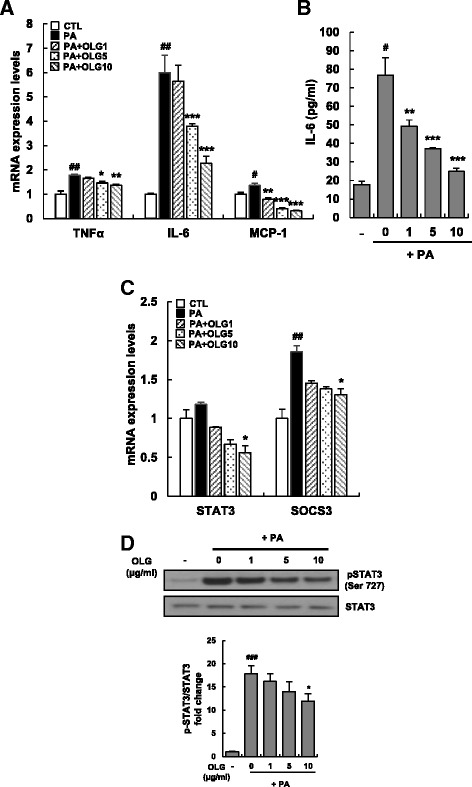
Oligonol suppressed inflammation responses induced by PA in HepG2 cells. HepG2 cells were treated with 250 μM of PA in the absence or presence of Oligonol (ranging from 1 to 10 μg/ml) for 24 h. (**a**) TNFα, IL-6, and MCP-1 mRNA levels were measured by qRT-PCR. (**b**) IL-6 released into the culture medium was assayed. (**c**) STAT3 and SOCS3 mRNA levels were measured by qRT-PCR. Values were normalized to GAPDH. Data were presented as mean ± SEM of three independent experiments; each performed in triplicates. *White bars* represent HepG2 cells treated with vehicle, and *black bars* represent HepG2 cells treated with PA. (**d**) STAT3 activation was examined by its phosphorylation state using Western blot analysis. β-actin was used as a loading control. Graphs represent densitometric analysis of Western blots. ^#^ *P* < 0.05, ^##^ *P* < 0.01, ^###^ *P* < 0.005 vs. vehicle-treated control (CTL); ^*^ *P* < 0.05, ^**^ *P* < 0.01, and ^***^ *P* < 0.005 vs. palmitate-treated cells (PA)

Chronic hepatic inflammation is tightly associated with the pathogenesis of NAFLD [24]. In fact, lipid peroxidation increases the production of inflammatory cytokines and mitochondrial dysfunction because β-oxidation generates increased levels of ROS, leading to oxidative stress [5, 25]. To determine the effect of Oligonol on the mRNA expression of inflammatory cytokines, HepG2 cells were treated with Oligonol in the presence of PA. The mRNA expression of genes encoding inflammatory cytokines tumor necrosis factor α (TNFα), interleukin 6 (IL-6), and monocytic chemotactic protein 1 (MCP-1) was significantly increased after PA treatment. However, Oligonol treatment at all the three concentrations decreased the expression of these inflammatory cytokines in a dose-dependent manner (Fig. 4a). The mRNA expression of IL-6 in PA-treated cells was ~6-fold higher than that in untreated cells. We measured the production and secretion of IL-6 by performing enzyme-linked immunosorbent assay (ELISA) and found that Oligonol decreased the production IL-6, which was consistent with the decreased mRNA levels of IL-6 (Fig. 4b).

Because IL-6 directly activates signal transducer and activator of transcription 3 (STAT3), the effect of Oligonol on STAT3 signaling was tested by examining the mRNA levels of STAT3 and suppressor of cytokine signaling 3 (SOCS3). PA did not increase the mRNA expression of STAT3. Further, Oligonol suppressed the mRNA expression of STAT3 by up to ~0.5 fold. PA markedly increased the mRNA levels of SOCS3 by ~2 fold, which exerts a negative feedback control in response to the activation of STAT3 (Fig. 4c). Western blotting analysis of phospho-STAT3 and STAT3 showed that PA increased the levels of phospho-STAT3 and that Oligonol decreased the levels of phospho-STAT3 at all the concentrations tested. Densitometric analysis showed no statistically significant decrease in the total level of STAT3. However, STAT3 level was decreased slightly, which was consistent with the decreased mRNA expression of STAT3 (Fig. 4d).

### Oligonol improved PA-induced insulin resistance

**Fig. 5:**
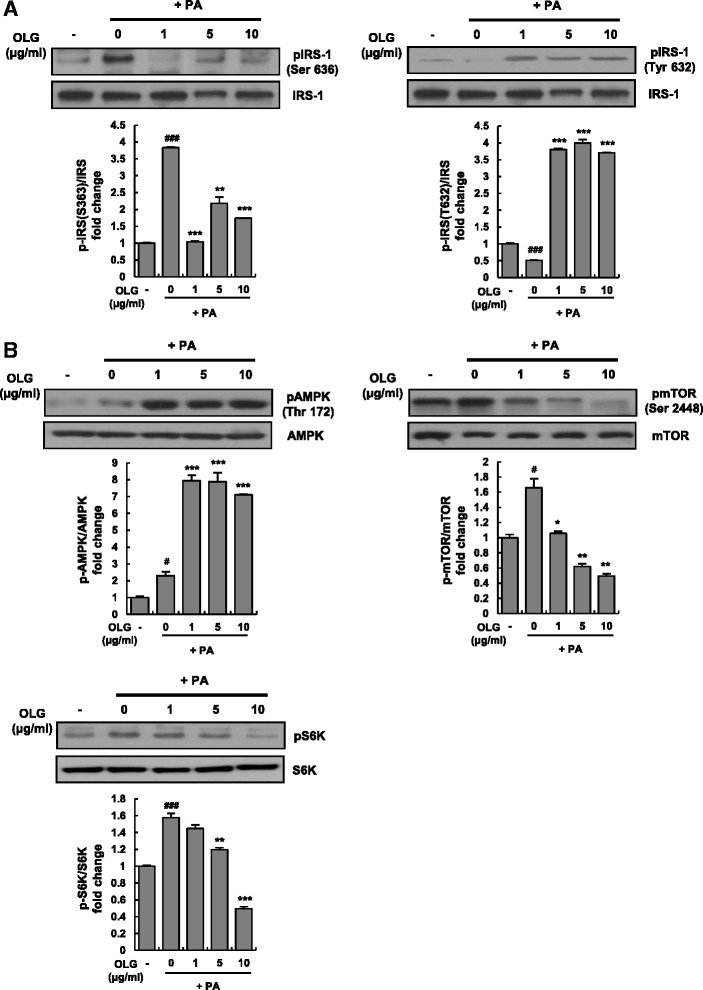
Oligonol attenuated PA-induced insulin resistance in HepG2 cells with concomitant activation of AMPK and suppression of the mTOR pathway. HepG2 cells were treated with 250 μM of PA in the absence or presence of Oligonol (ranging from 1 to 10 μg/ml) for 24 h. The phosphorylation states of (**a**) IRS-1 or (**b**) AMPK, mTOR, and S6K were examined by Western blot analysis with appropriate antibodies. β-actin was used as a loading control. Graphs represent densitometric analysis of Western blots. ^#^ *P* < 0.05, ^##^ *P* < 0.01, ^###^ *P* < 0.005 vs. vehicle-treated control (CTL); ^*^ *P* < 0.05, ^**^ *P* < 0.01, and ^***^ *P* < 0.005 vs. palmitate-treated cells (PA)

Based on our finding that Oligonol suppressed IL-6–STAT3–SOCS3 signaling in PA-treated HepG2 cells, we examined the effect of Oligonol on the key proteins involved in insulin signaling. We examined the changes in the phosphorylation of insulin receptor substrate-1 (IRS-1) in PA-treated HepG2 cells to assess insulin resistance. IRS-1 activity is either activated or inhibited by the phosphorylation of specific tyrosine or serine residues [26]. We first confirmed that PA treatment increased IRS-1 phosphorylation at serine 636 (an inhibitory site for insulin signaling) and decreased IRS-1 phosphorylation at tyrosine 632 (a stimulatory site for insulin signaling). Interestingly, Oligonol treatment reversed this phosphorylation pattern, i.e., phosphorylation at serine 636 was attenuated while that at tyrosine 632 was increased by all the 3 concentrations of Oligonol (Fig. 5a). To further confirm that insulin resistance was relieved in these cells, we examined the expression of genes involved in insulin resistance (*INSR*, *CREB*, *CRTC*, *IGFBP-1*, and *GLUT2*). No significant differences were observed in the expression of these genes between cells treated with Oligonol plus PA and untreated control cells (data not shown).

### Oligonol activated AMPK and inhibited mTOR–S6K pathway in HepG2 cells

AMP-activated protein kinase (AMPK) is a critical enzyme for regulating hepatic lipogenesis and insulin resistance. Activated AMPK increases fatty acid oxidation, lipolysis, and insulin sensitivity in the skeletal muscle, liver, and adipose tissue [18, 27]. To determine the modulation of AMPK by Oligonol, AMPK activation was examined by analyzing its phosphorylation levels in total cell lysates by Western blot analysis. All the concentrations of Oligonol equally enhanced the activity of AMPK, as determined by phosphorylation at threonine 172 compared with that in untreated control or PA-treated cells. Because AMPK negatively regulates mammalian target of rapamycin (mTOR) [28], we investigated whether Oligonol suppressed the activation of mTOR and its downstream component ribosomal protein S6 kinase (S6K). Western blotting analysis of mTOR and S6K showed that Oligonol markedly suppressed the phosphorylation of both mTOR and S6K in a dose-dependent manner. No differences were observed in the total protein levels of AMPK, mTOR, and S6K (Fig. 5b).

## Discussion

Many polyphenols exert anti-obesity and hypolipidemic effects in animals and humans. These compounds have been investigated as complementary agents because of their potential beneficial effects on health and biosafety in various model systems [6, 29]. In the present study, we observed that Oligonol inhibited de novo fatty acid synthesis in HepG2 cells. We also found that Oligonol suppressed the expression of inflammatory cytokines such as IL-6, thus exhibiting anti-inflammatory effects, and regulated insulin resistance through the STAT3–SOCS3 and AMPK pathways. In the present study, we used PA, which is widely used to establish hepatic steatosis models that show increased FFA production [7]. Fatty acid metabolism in the liver involves the following steps: (1) de novo synthesis of fatty acids and uptake of plasma FFAs; (2) fatty acid oxidation in the mitochondria, peroxisomes, and microsomes; (3) neutralization of ROS generated through fatty acid oxidation; and (4) conversion of TGs to fatty acids [21]. Oligonol suppressed both ACC and FAS, which are the primary enzymes in lipogenesis. Oligonol also suppressed the mRNA expression of SREBP-1 and PPARγ, indicating that this anti-lipogenic effect occurred at the transcriptional level. Lipogenesis is regulated by SREBP-1 and SREBP -2. Particularly, SREBP-1 positively controls FFA metabolism by inducing the expression of target genes encoding rate-limiting enzymes such as ACC and FAS involved in de novo lipogenesis [30]. Oligonol activated the enzymes involved in lipid metabolism. Oligonol increased the phosphorylation of ACC and HSL (Fig. 2c). Phosphorylation of ACC at serine 79, a key phosphorylation site, inactivates ACC [31]. Thus, phosphorylation of ACC at serine 79 is a critical step in lipogenesis, indicating that this enzyme is a potential target for treating dyslipidemia [32]. Oligonol acts as a regulatory checkpoint in lipid synthesis by increasing phosphorylation. In fact, the mRNA expression of ACC, FAS, and SREBP-1 is increased in the livers of patients with NAFLD [21]. Moreover, liver-specific ACC1-knockout (LACC1KO) mice show decreased de novo concentrations of fatty acids and hepatic TGs [33].

Oligonol contributes to lipolysis by simultaneously inhibiting adipogenesis. Oligonol increases the release of glycerol from differentiated 3T3-L1adipocytes [34]. Moreover, Oligonol reduces fat content in the adipose tissues by increasing lipolysis both in vitro and in vivo [35, 36]. Consistent with previous studies, Oligonol exerted a lipolytic effect on HepG2 cells in the present study, suggesting that Oligonol can efficiently regulate lipid metabolism in the adipose tissues and liver to prevent dyslipidemia. We further investigated the effect of Oligonol on the expression of genes associated with β-oxidation. Oxidation of fatty acids occurs mainly in the mitochondria. However, excessive fatty acid production in hepatocytes also activates alternative pathways such as β-oxidation in the peroxisomes and ω-oxidation in the microsomes. In patients with NAFLD, mRNA expression of genes involved in fatty acid oxidation, such as *LCAD*, *HADHα*, *CYP2E1*, and *CYP4A11*, is increased while that of *CPT1A* and *PPARA* is decreased compared with that in the livers of normal individuals [21, 37]. PA, which is widely used to establish an in vitro model of fatty liver, mimicked dyslipidemic conditions by decreasing the mRNA levels of CPT1a and increasing the mRNA levels of CYP2E1 and CYP4A11. However, PA did not increase the mRNA levels of LCAD and HADHα. Moreover, mRNA levels of HADHα increased after Oligonol treatment in a dose-dependent manner. Because Oligonol reduced the mRNA levels of CYP2E1 and CYP4A11, which were highly induced by PA, it is possible that acute PA stimulation may disrupt mitochondrial β-oxidation and alternatively induce ω-oxidation. Thus, Oligonol reversed PA-induced expression of the above genes, suggesting that Oligonol facilitates the transport of long-chain fatty acids into the mitochondria and restores mitochondrial β-oxidation by increasing the mRNA expression of CPT1a and HADHα.

Oligonol appears to eliminate excessive amounts of reactive oxygen species (ROS) produced during lipid oxidation in the mitochondria. UCP2 is a mitochondrial inner membrane carrier protein that mediates a proton leak across the inner membrane and uncouples mitochondrial electron transport from adenosine triphosphate (ATP) synthesis [38]. In many pathophysiological conditions such as NAFLD and obesity, UCP2 levels are elevated, indicating increased oxidative stress [6, 39]. Our results showed that PA-induced UCP2 levels were inhibited by Oligonol, suggesting that Oligonol functions as an antioxidant and decreases the levels of ROS in HepG2 cells. However, this result was not consistent with the observation that Oligonol increased the gene expression of *CPT1A* and *HADHα* and enhanced β-oxidation because increased β-oxidation induces ROS production. Therefore, it is conceivable that UCP2 levels may increase upon Oligonol treatment. However, UCP2 is not normally expressed in hepatocytes [40]; thus, the role of UCP2 in hepatocytes should be elucidated in further studies. Transcription of *UCP2* is regulated by other conditions such as energy expenditure and inflammation.

On the other hand, increase in the levels of inflammatory cytokines is associated with UCP2 expression. Incubation of hepatocytes with FFA increases the production of ROS and mRNA level of UCP2 [41], leading to oxidative stress. Overproduction of ROS also activates nuclear factor kappa beta (NF-kB) [42], a major transcription factor, that upregulates the expression of various cytokine such as TNFα and IL-6 and contributes to mitochondrial dysfunction and increased ROS production. In fact, serum and hepatic levels of TNFα, IL-1, and IL-6 were elevated in a mouse model of NAFLD and patients with NAFLD [43–45]. Conversely, anti-cytokine antibodies decreased inflammation and prevented dyslipidemia and NAFLD in a mouse model [46, 47]. Thus, it is possible that PA induces acute inflammation and expression of TNFα and IL-6 in HepG2 cells. The above data indicate that these cytokines and not β-oxidation increase ROS production, leading to the upregulation of *UCP2* mRNA levels. Thus, Oligonol may act as an antioxidant or anti-inflammatory agent to reduce the levels of UCP2.

Many studies have shown that hepatic steatosis is tightly associated with insulin resistance and obesity. A previous study reported that 40–100 % obese people have comorbidities such as NAFLD and that 21–55 % obese people have type 2 diabetes or hyperglycemia [48]. Increased levels of inflammatory cytokines play an important in the development of insulin resistance. TNFα was the first cytokine to be identified that impaired insulin action [49]. In obese rodent models and humans with NAFLD or obesity, a strong inverse correlation has been observed between serum TNFα levels and insulin-stimulated glucose metabolism [50, 51]. Although mechanisms involved in TNFα-induced insulin resistance are not completely understood, compelling evidence indicates that TNFα inhibits IRS-1- and IRS-2-mediated PI3-kinase activation, leading to insulin resistance [52]. IL-6 is another important cytokine involved in the regulation of insulin resistance. IL-6 promotes insulin resistance by activating the STAT3–SOCS3 pathway in the liver [53]. Activation of STAT3 by phosphorylation subsequently increases SOCS3 expression through a negative feedback mechanism. SOCS3 then directly disrupts insulin signaling through ubiquitin-mediated degradation of IRS [18, 54]. In the present study, Oligonol modulated reciprocal phosphorylation of IRS-1 by increasing the phosphorylation of the stimulatory residue tyrosine 632 and by decreasing the phosphorylation of the inhibitory residue serine 636, thus restoring insulin signaling. We further examined the expression levels of genes involved in insulin resistance. However, no changes were observed in the expression of these genes. To further understand the effects of Oligonol, a study involving a high-fat-induced obese mouse model should be performed to determine the comprehensive role of Oligonol in lipid metabolism.

In our previous study, Oligonol inhibited TG accumulation and lipogenesis by suppressing Akt–mTOR pathway in 3T3-L1 adipocytes [34]. Therefore, we tested whether Oligonol had the same anti-lipogenic effect on hepatic steatosis by using PA-treated HepG2 cells. Activated AMPK inhibits ATP-consuming processes such as synthesis of fatty acids or sterols and activates ATP-producing processes such as fatty acid oxidation [55]. Once activated through phosphorylation at threonine 172 residue in its α subunit, AMPK inhibits hepatic lipogenesis by phosphorylating ACC at the inhibitory serine 79 residue and by downregulating SREBP-1c [55]. Because Oligonol suppresses lipogenic gene expression and ACC activation (Fig. 2), we hypothesized that Oligonol activates AMPK and consequently inhibits lipogenic processes. As expected, Oligonol potently activated AMPK at all the tested concentrations and subsequently inhibited mTOR activation, thus inhibiting IRS-1 [26]. Interestingly, an anti-diabetic agent metformin, which activates AMPK in hepatocytes, exerted beneficial effects on lipid metabolism by suppressing the activity of ACC and reducing the expression of lipogenic enzymes [56]. Thus, the AMPK–mTOR pathway is a potential therapeutic target for treating hepatic steatosis. Oligonol showed a similar mechanism of action, indicating its beneficial effects in preventing insulin resistance and hepatic steatosis. IL-6 plays a paradoxical role in insulin signaling, which improves insulin sensitivity after physical exercise. Ruderman et al. reported that IL-6 treatment of both muscle cells and adipocytes increased AMPK and ACC phosphorylation. Similar findings were observed in the muscle of mice after exercise. Moreover, these effects were decreased in IL-6 knockout mice [57]. IL-6 treatment improved insulin-induced glucose disposal and was accompanied by increased glucose transporter-4 (GLUT4) translocation to the plasma membrane and activation of AMPK [58]. However, IL-6-induced AMPK activation appears to be an acute response in skeletal muscle tissues after exercise. Low-grade chronic elevation of IL-6 is the primary cause of NAFLD. Thus, the prolonged effect of IL-6 on AMPK needs to be investigated further in hepatic steatosis models.

## Conclusions

NAFLD is one of the most common causes of liver dysfunction. Excessive FFAs in the liver induce oxidative stress, inflammatory cytokine production, mitochondrial dysfunction, and insulin resistance, which all contribute to disease progression. In this study, we provided evidence that Oligonol has a protective effect on PA-induced cellular steatosis in HepG2 cells. Oligonol suppressed lipogenesis, while promoting fatty acid oxidation and lipolysis. PA-induced IL-6 production and STAT3 phosphorylation were significantly repressed by Oligonol. In addition, Oligonol inhibited mTOR phosphorylation by AMPK activation. Our results demonstrated that Oligonol may prevent hepatic steatosis by regulating AMPK-mTOR and STAT3-SOCS3 signaling pathways, and that these pathways are good therapeutic targets for treating hepatic dysfunction.

## Abbreviations

NAFLD: Non-alcoholic fatty liver disease
NASH: Non-alcoholic steatohepatitis
TG: Triglycerides
PA: Palmitate
FFAs: Free fatty acids
ACC: Acetyl-CoA carboxylase
FAS: Fatty acid synthase
SREBP-1: Sterol regulatory element-binding protein-1
PPARγ: Peroxisome proliferator-activated receptor-γ
HSL: Hormone-sensitive lipase
CPT1α: Carnitine palmitoyltransferase 1a
LCAD: Long-chain acyl-CoA dehydrogenase
HADHα: Long-chain L-3-hydroxyacyl-coenzyme A dehydrogenase α
UCP2: Uncoupling protein 2
PPARα: Peroxisome proliferator-activated receptor-α
TNFα: Tumor necrosis factor α
IL-6: Interleukin 6
MCP: Monocytic chemotactic protein 1
STAT3: Signal transducer and activator of transcription 3
SOCS3: Suppressor of cytokine signaling 3, IRS-1, insulin receptor substrate-1
AMPK: AMP-activated protein kinase
mTOR: Mammalian target of rapamycin
S6K: Ribosomal protein S6 kinase
ROS: Reactive oxygen species
ATP: Adenosine triphosphate
NF-kB: Nuclear factor kappa beta
GLUT4: Glucose transporter-4
